# Neutron tomography of sealed copper alloy animal coffins from ancient Egypt

**DOI:** 10.1038/s41598-023-30468-4

**Published:** 2023-04-20

**Authors:** Daniel O’Flynn, Anna Fedrigo, Laura Perucchetti, Aurélia Masson-Berghoff

**Affiliations:** 1 Department of Scientific Research, The British Museum, Great Russell Street, London, WC1B 3DG UK; 2 Science and Technology Facilities Council (STFC), ISIS Neutron and Muon Source, Didcot, OX11 0QX UK; 3 Department of Greece and Rome, The British Museum, Great Russell Street, London, WC1B 3DG UK

**Keywords:** Applied physics, Techniques and instrumentation, Imaging techniques

## Abstract

Animal mummification was commonplace in ancient Egypt, with the remains of many animals placed inside statues or votive boxes with representations of animals or hybrid human–animal creatures. Votive boxes were made from a variety of materials and often sealed; some boxes are still preserved in this state in museum collections. A prior study of sealed copper alloy votive boxes from the collection of the British Museum used X-ray computed tomography to search for animal remains, where poor image quality resulted due to attenuation from the boxes and apparent dense metals inside. In this study, neutron tomography was applied to six of the votive boxes previously examined. Animal remains, likely from lizards, and fragments of textile wrappings were discovered inside three of the boxes. Evidence of the manufacturing process and subsequent repairs of the boxes were uncovered by neutrons. Significant quantities of lead were also identified in three boxes. The findings demonstrate the effectiveness of neutron tomography for the study of mummified remains inside sealed metal containers, and give evidence linking the animal figures represented on top of votive boxes to the concealed remains.

## Introduction

The mummification of animals was a widespread practice in ancient Egypt. Animal remains, believed to be physical incarnations of deities, votive offerings or part of a ritual performance, have been discovered inside many religious complexes, mostly dating to the 1st millennium BCE^1–4^. Remains were sometimes placed within statues of animals or inside boxes featuring a representation of the animal on the top^1,4–8^. Such boxes are interchangeably referred to in Egyptological literature as ‘animal coffins’ or ‘votive boxes’, although it is not always clear if they systematically contain the remains of an animal, or if they were votive in nature rather than performing some ritual function^9^. Many animal species were depicted on the boxes, including falcons, cats, mongooses, snakes, eels, lizards, and shrews. Different materials were used in the manufacture of the boxes, including wood, limestone, and copper alloy (notably bronze or leaded bronze^10,11^), and they vary widely in their shape and size.

A small limestone box topped by a shrew figure, discovered in Saqqara, was found to contain a shrew mummy using X-radiography^8^. No remains of wrappings were discovered in the box, although their original presence could not be discounted. X-radiography has also been used to uncover a snake mummy inside a wooden box topped by a snake figure^7^.

Copper alloy votive boxes were typically made by casting, with an opening at one end which was subsequently sealed with a plaster plug and metal panel. Many of these boxes were not intact when discovered, and typically no animal remains were present inside. Fragments of cat bone were found inside an opening in a bronze box with a seated bronze cat figure on top^12^ (British Museum EA65795). In some cases, only pieces of textile were found inside the boxes, possibly the remains of animal wrappings^1^.

X-ray computed tomography (CT) has been successfully applied to the non-invasive study of wrapped and unwrapped animal mummies, using medical CT scanners, laboratory-based microfocus X-ray CT systems, and with synchrotron microtomography^13–15^. A limitation of X-ray imaging is the presence of metal—particularly lead or leaded bronze—or other very dense materials in the beam path. X-ray attenuation by dense objects leads to image artefacts in reconstructed X-ray CT volumes, such as streaking and beam hardening^16,17^. These artefacts can obscure features of interest, particularly those in lower density materials.

A previous study applied X-radiography and X-ray CT to a group of eight intact copper alloy votive boxes in the British Museum collection, topped by depictions of eels, reptiles, and human–eel–cobra hybrids^9^. X-ray CT scans gave evidence for animal bones inside some of the boxes, although the image quality was hampered by strong X-ray attenuation from the metal, despite the use of X-ray tube voltages up to 450 kV. Indications of the manufacturing process of the boxes and the methods used to seal the open ends were observed in both X-radiography and X-ray CT. Inside three of the boxes, very dense objects were identified from the intense streaking artifacts in the data. These were suspected to be made from copper alloy or lead, but no further detail could be revealed due to their thickness and density.

Neutron imaging has been established as a complimentary non-invasive technique to X-ray imaging^18,19^. Whilst X-rays are generally more strongly attenuated by elements with a greater atomic number, neutrons show no such correlation; notably, in contrast to X-rays, neutrons are strongly attenuated by hydrogen and weakly attenuated by metals, including lead. Neutron imaging can therefore be particularly effective for detecting organic material, and light materials more generally, enclosed within dense casings—for example, water inside porous rock^20^. These properties have successfully been applied to cultural heritage studies: revealing the organic content of bronze Tibetan Buddha statues^21,22^ and relics inside an altar stone^23^; examining the manufacture of a bronze ship model^24^; and mapping corrosion phases in iron swords^25^. Neutron tomography has also been compared with X-ray CT in the study of a wrapped cat mummy^26^. In a 1985 study by Jett, Sturman, and Drayman-Weisser, a bronze falcon statue in the Walters Art Museum, Baltimore, was found to contain bird bones by use of an endoscope inserted through a small opening in its head. X-radiography was unable to reveal further information on the remains due to strong X-ray attenuation from the bronze; neutron radiography gave a much-improved image of the bones inside the statue^6^.

This article details the neutron tomography of six copper alloy votive boxes from the British Museum collection previously studied with X-radiography and X-ray CT imaging^9^. These rare examples of still-sealed boxes were selected based on the potential presence of interesting features/material within suggested by the previous study. There is a debate among Egyptologists regarding the nature and function of such boxes, hence there is an interest in checking for the presence or absence of animal mummies within. Many boxes are very small and would not have been able to accommodate the body of the mummified animal represented on top, or at least not the complete body. It is also difficult to prove that these boxes were exclusively votive objects. Recent discussions regarding mass-produced Egyptian bronzes—which are contemporaneous to this group of six boxes, and often found in similar archaeological contexts—suggest that some could have been used during rituals^27^, which may have also been the case for some of these boxes. Building on the past work, the aims of the experiment were to identify organic remains inside the boxes, to gain insight into the box manufacture, and to identify the unknown dense objects previously observed inside three of the boxes.

## Votive boxes

Three of the votive boxes examined in this study—British Museum accession numbers EA27584, EA49144, and EA49146—were discovered in Naukratis in the western Nile Delta in 1885. Naukratis was an international harbour founded in the late seventh century BCE and was a key part of a trade network between the Mediterranean world and the Nile Valley. The boxes feature depictions of lizards and eels, and are thought to date from 500 to 300 BCE^2,4,28^.

Box EA36167, topped by a lizard figure, was discovered in Tell el-Yehudiyeh in the eastern Nile Delta and was purchased by the British Museum in 1876. The box is attributed to the Late Period (664–332 BCE), and no further information is known on its findspot or context. A narrow crack runs along the base of the box, but it does not appear to go all the way through to the interior.

Boxes EA71428 and EA36151 (each with unknown provenance) are both topped with a part-eel, part-cobra figure, with a human head. Box EA71428 was registered into the British Museum collection in 1989 and box EA36151 was purchased by the Museum in 1867. They are both likely dated to the Late Period, early Ptolemaic period at the latest, mid-seventh to third century BCE. Votive boxes with depictions of eels and lizards were associated in ancient Egypt with the solar and creator god Atum^29^. Atum is often represented in anthropomorphic form, as a human-headed part-eel, part-cobra creature wearing a double crown.

All six votive boxes studied are made from copper alloy, and are still sealed by a plaster plug; a drill hole is present in the plug of box EA49144, although it does not fully penetrate it. Details and photographs of the six boxes are given in Table 1.

**Table 1:** Descriptions of the boxes investigated in this study (images not to scale). Photographs © The Trustees of the British Museum, and shared under a CC-BY-NC-SA 4.0 license.

Accession Number	Image/s	Size (mm)	Findspot	Description of box exterior
EA27584		29 × 32 × 55	Naukratis	Two lizard figures, single suspension loop
EA49144		19 × 14 × 78	Naukratis	Eel figure, two suspension loops. Drill-hole in sealed opening does not fully penetrate plug
EA49146		22 × 17 × 55	Naukratis	Lizard figure, two suspension loops
EA36167		16 × 26 × 90	Tell el-Yehudiyeh	Lizard figure detailed with dots and lines on skin
EA71428		64 × 25 × 187	Egypt	Human-headed eel-cobra creature
EA36151		146 × 40 × 298	Egypt	Human-headed eel-cobra creature

## Results

In the results detailed below, the following convention has been used for the orientation of tomographic slices: right/left—corresponding with the proper right/left of the creature surmounting the box; front—the end of the box nearest to the head of the creature (opposite the sealed opening). Top view slices are oriented such that the right wall is at the bottom and the front wall is to the right of the slice. Side view slices are oriented such that the box base is at the bottom and the front wall is to the right of the slice. Front view slices are viewed from the front of the box, such that left and right walls are mirrored. The uncertainties in distance measurements are ± 0.2 mm for the first five boxes listed (0.055 mm reconstructed voxel size), and ± 0.4 mm for box EA36151 (0.103 mm voxel size).

### EA27584

**Figure 1:**
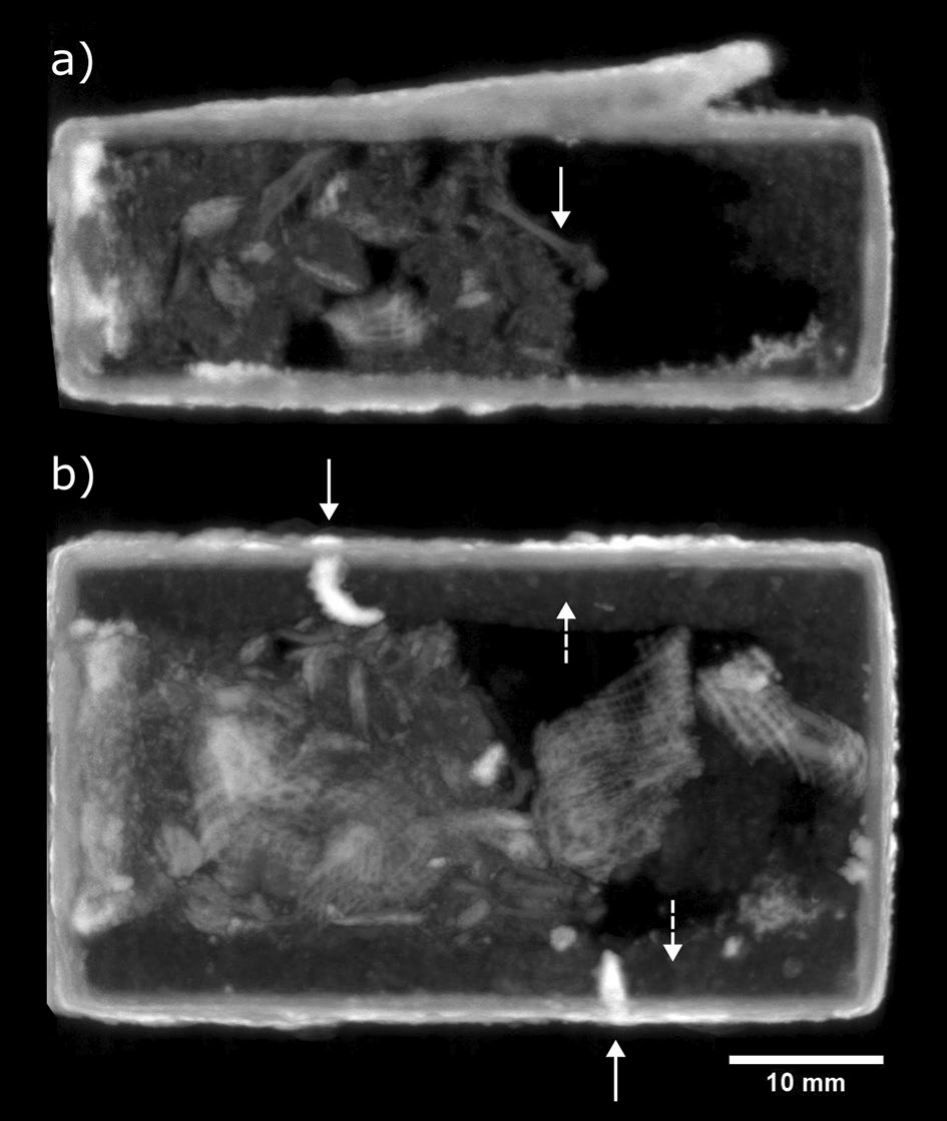
EA27584 multiplanar maximum intensity projection (MIP) neutron CT images: (**a**) side-view, 1.16 mm thick slice centred 6.1 mm from right side of box, highlighting the presence of an 8.1 mm long bone (solid arrow); (**b**) top-view, 12.43 mm thick slice centred 8.3 mm from base of box, showing the presence of textile, corroded chaplets (solid arrows), and core material (dashed arrows).

The box topped by two lizards was found to contain animal remains and textile pieces seemingly used to wrap the remains prior to their placement in the box (Fig. 1). Although the animal remains are in a fragmentary condition, a long bone measuring approx. 8.1 mm can be seen (Fig. 1a). The textile could be linen, cotton or wool^30^, but is thought to be linen, since it is commonly used in animal mummy wrappings^31^. The textile has a loose weave, with 1–2 mm spacing between the threads. Evidence of the lost-wax casting technique^32^ can be seen in the presence of chaplets and a layer of core material (possibly clay) covering the internal walls (Fig. 1b). The chaplets are embedded in the box walls and core material and are strongly attenuating of neutrons (linear attenuation coefficients of 1.5–1.6 cm^−1^), likely due to hydrogen-containing metal corrosion products such as hydroxides. The significantly higher amount of corrosion on the chaplets suggests they were made of iron, since iron is more likely to oxidise than copper alloys, and thus corrodes more quickly. Further evidence of corrosion is visible on the outer layers of the box and lizard figures. There is no visible discontinuity between the box and the loops or lizard figures, suggesting that all were manufactured in a single casting. Neutron CT volume renders of box EA27584 are given in Supplementary Information Fig. S1.

### EA49144

**Figure 2:**
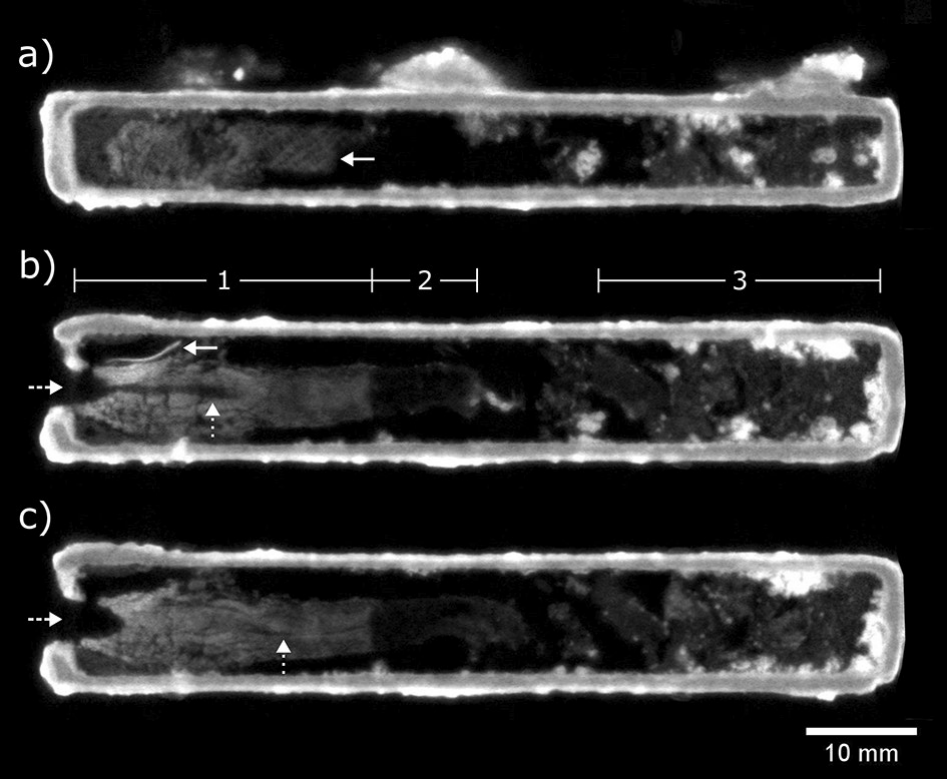
EA49144 neutron CT slices. (**a**) Side-view, slice 3.2 mm from right side of box (solid arrow: textile in plug). Top-view slices at (**b**) 4.0 mm and (**c**) 5.3 mm from base of box, with internal regions (**1**) plaster plug (**2**) weakly-attenuating end of plug, (**3**) fragments. Solid arrow: unidentified object adjacent to plug; dashed arrows: drilled hole in plug; dotted arrows: narrow void extending through plug.

Neutron tomography of the box topped by the eel figure and two suspension loops revealed the presence of a plaster plug on one side and fragments and concretions on the opposite end, but no identifiable animal remains (Fig. 2). The most interesting feature is the presence of a textile fragment in the plaster plug, clearly showing the individual threads, spaced approx. 0.8 mm apart and arranged in a plain weave, (i.e., the warp and weft threads cross at right angles) (Fig. 2a). The plug continues up to 46 mm into the box. There is a sharp drop in the neutron attenuation of the plug from approx. 0.3–0.1 cm^−1^ beyond a depth of 27 mm (at the boundary between denoted regions 1 and 2 in Fig. 2b,c). It is not clear what is the cause of this difference in attenuation; one possible explanation is that the plaster in the plug only extends to this depth, and beyond is only the more weakly-attenuating textile. The 3.5 mm wide drill hole in the plug, visible from outside the box, extends approx. 5 mm into the plug. Another, narrow void running along the length of the plug, was revealed by neutron CT. This void is not straight, and varies from 0.2 to 0.9 mm width, suggesting that it is not a drill hole, but perhaps a gap from the folding of the textile inside the plug.

The fragmented material in the box comprises a matrix containing multiple rounded objects up to 1.2 mm in width with neutron attenuations ranging from 0.6 to 0.9 cm^−1^. An additional unidentified object is present inside the box, close to the plug (Fig. 2b). The object is approx. 7.7 × 1.5 × 0.3 mm in size, with the shape of a curled piece of paper or fabric, and has a neutron attenuation of 0.9–1.0 cm^−1^.

### EA49146

**Figure 3:**
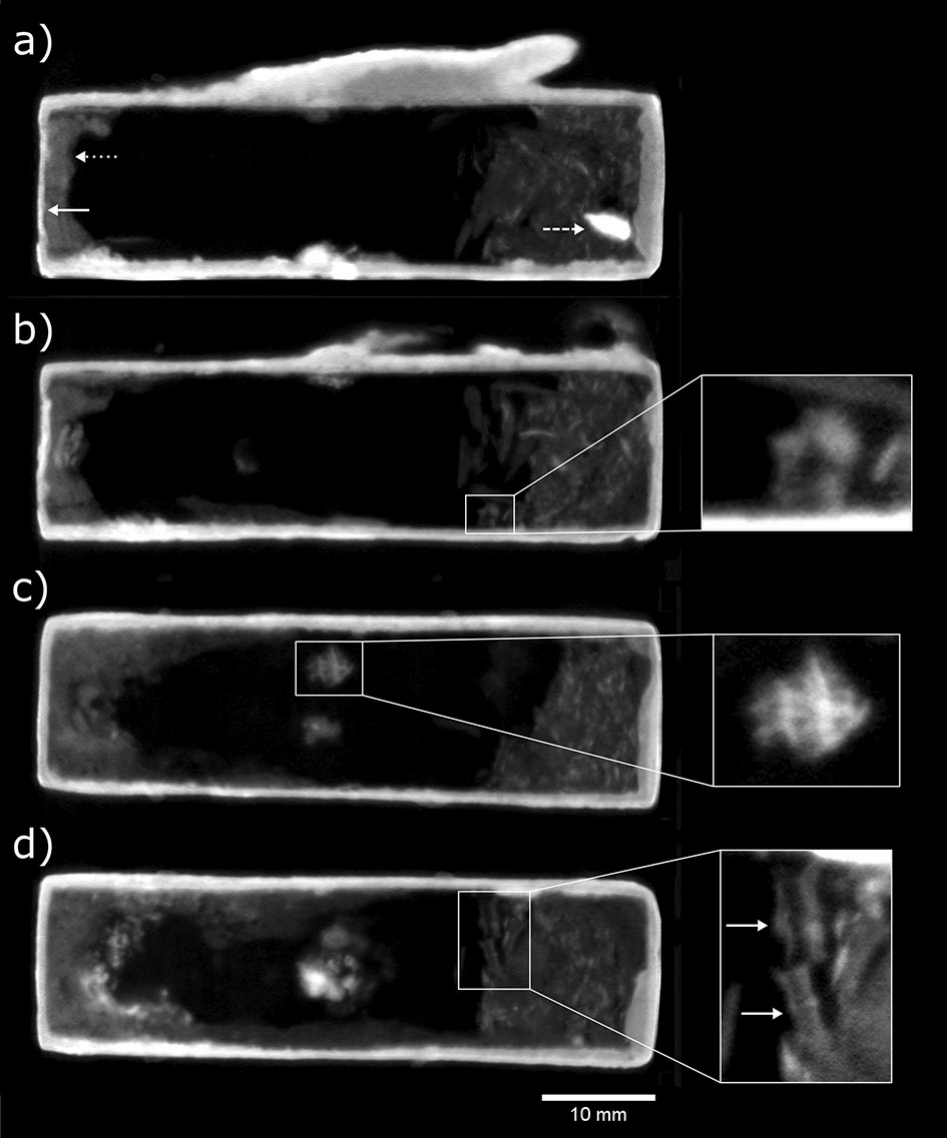
EA49146 multiplanar neutron CT slices, averaged over 0.44 mm slice thickness: (**a**) side-view slice centred 7.0 mm from right side of box (solid arrow: metal plate covering plug, dotted arrow: plug, dashed arrow: loose chaplet); (**b**) side-view slice centred 11.7 mm from right side of box (inset: vertebra, transverse cross-section); (**c**) top-view slice centred 13.8 mm from base of box (inset: textile fragment); (**d**) top-view slice centred 2.1 mm from base of box (inset: vertebrae, longitudinal cross-section, indicated by solid arrows).

Several small vertebrae measuring approx. 1 × 1.5 × 2 mm were revealed by the neutron CT of box EA49146 (Fig. 3), as well as multiple loose fragments, possibly bone. The neutron attenuation coefficients of the vertebrae and the fragments were measured to be 0.3–0.5 cm^−1^, comparable with the long bone in box EA27584 (0.4–0.5 cm^−1^). A small textile fragment was seen near the top of the box interior, measuring approx. 3 × 3 mm. Three corroded chaplets are present, one of which has broken from the right wall and is lying loosely inside the box.

### EA36167

**Figure 4:**
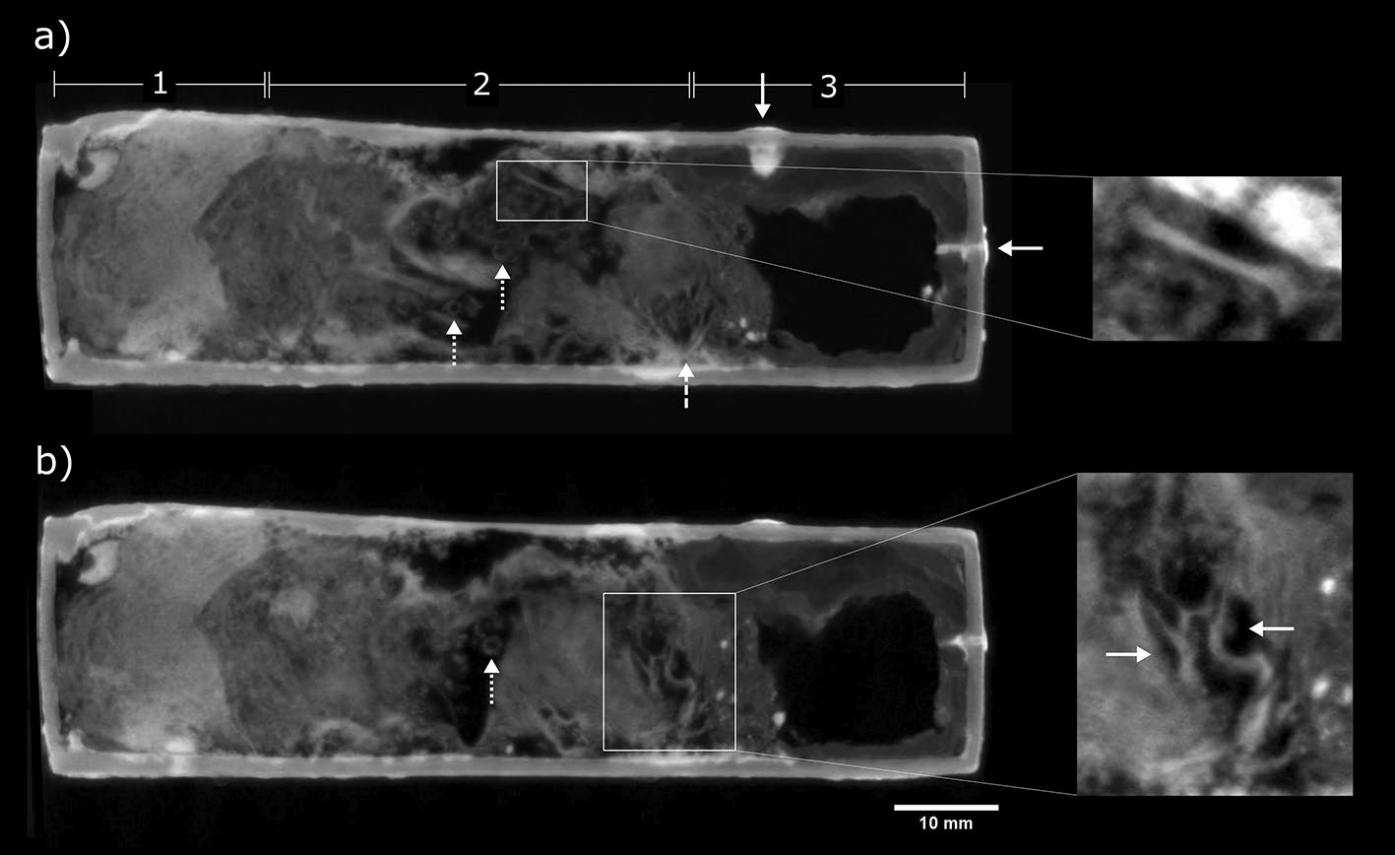
EA36167 multiplanar neutron CT slices, averaged over 0.55 mm slice thickness: (**a**) top-view slice centred 6.9 mm from base of box showing three internal regions, (**1**) plaster plug sealing the box, (**2**) textile and animal remains, (**3**) lead with void in centre (solid arrows: corroded chaplets; dotted arrows: vertebrae, transverse cross-sections; dashed arrow: lizard skull; inset: contrast-enhanced view of a long bone measuring 6.2 mm in length); (**b**) top-view slice centred 8.9 mm from base of box (dashed arrow: vertebra; inset: contrast-enhanced view of upper part of lizard skull in top-view, with orbits indicated by solid arrows).

Box EA36167 has different regions and various materials throughout its interior. Immediately behind the metal panel sealing the opening is a plaster layer of 13–20 mm length, followed by a textile bundle measuring approximately 50 × 20 × 11 mm. Beyond the bundle, at the end of the box furthest from its opening, is a low-attenuating material at the interface with the box walls, with a void in its centre (Fig. 4). Three corroded chaplets—showing as bright areas—are present inside the walls of the box, one in the wall opposite the opening, and one in each of the side walls.

Within the textile bundle there seem to be several small bones and bone fragments, including a complete long bone of 6.2 mm length, and vertebrae measuring approx. 1.5 × 1.5 × 2.0 mm. Towards the end of the bundle furthest from the box opening appears to be an intact lizard skull, with mandible and orbits seen in top-view CT slices (Fig. 4). The mandible is approx. 8.5 mm wide × 10.4 mm long, and the orbits approx. 1.8 mm wide × 3.1 mm long. Although it is not possible to identify the species of lizard from the neutron tomography data due to the variability of sizes within species, the sizes of the bones are consistent with lizards of the *Mesalina* genus, several species of which are endemic to northern Africa^33–35^. The lizard figure on top of the votive box is decorated with spots and stripes running along the length of the back; several species of the *Mesalina* genus are also spotted and/or striped. An X-ray micro CT scan of *M. rubropunctata* was accessed on the MorphoSource repository^36^, for comparison with the remains found inside EA36167; the sizes of the orbits, mandible, C1 vertebra and long bones of this specimen are given in Supplementary Information Table S1, and are broadly similar to those measured from the neutron CT scan of the votive box.

The material at the end of the box furthest from the opening can be identified as lead due to its low neutron attenuation compared with strong X-ray attenuation previously reported in this region^9^. Based on the shape of the lead, and the fact that it surrounds two chaplets, it is assumed that the lead was introduced to the box in a molten state. Given the void in the lead, we cannot exclude that something was originally inside. A small region of concretion, possibly clay/soil with mineral inclusions, is seen inside the lead, in contact with the textile bundle. Regions of strong attenuation, due to corrosion, are present in the lead at opposite ends of the void (see Supplementary Information Fig. S2). A potential cause of this corrosion is decaying animal matter in proximity with the lead.

### EA71428

**Figure 5:**
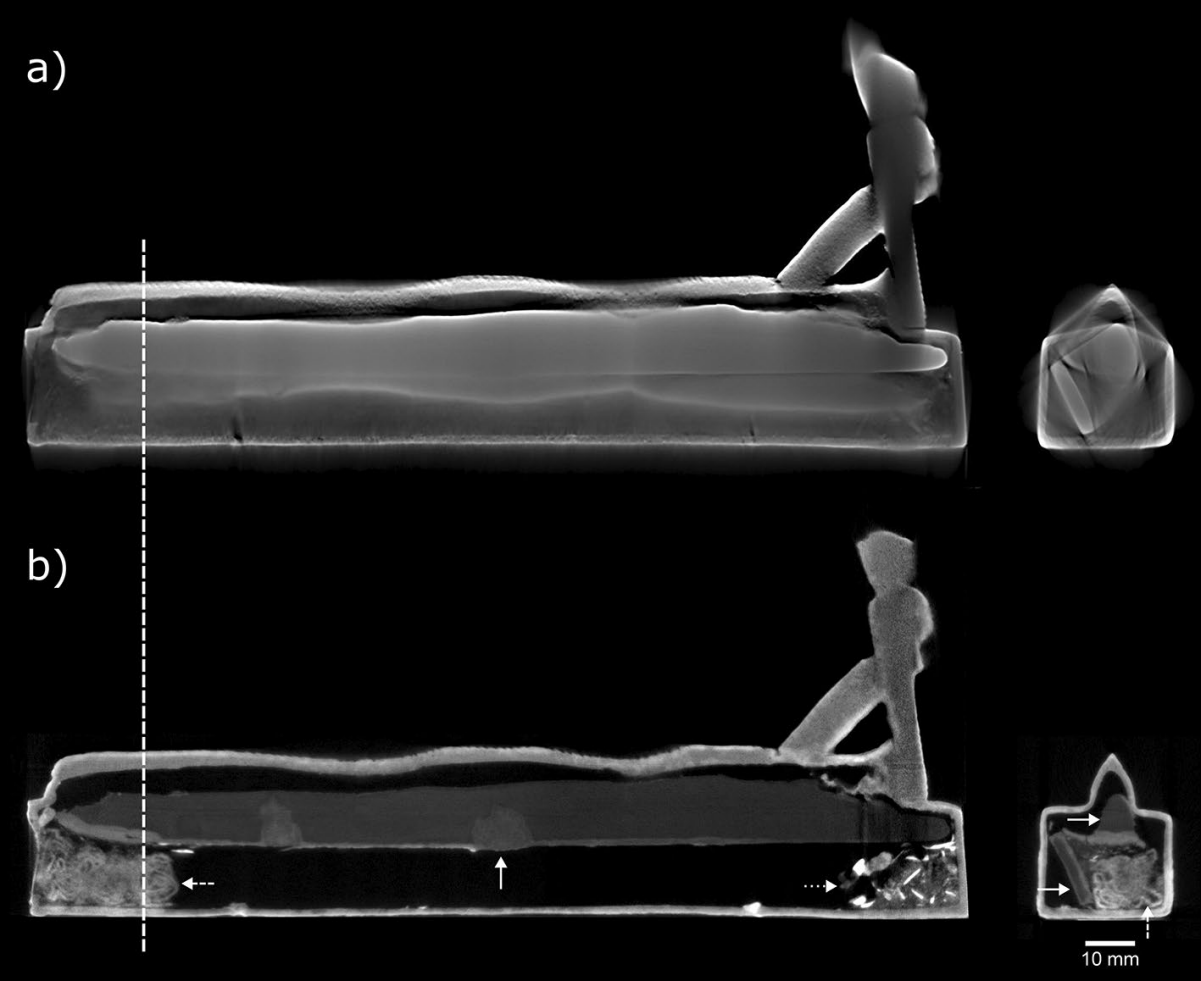
EA71428 (**a**) X-ray and (**b**) neutron CT slices (side-view and front-view). Front-view slices taken at the position marked by the dashed line. The neutron CT images are averaged over two adjacent planes (giving a 0.110 mm slice thickness), to approximately match the X-ray CT data (0.108 mm slice thickness). Solid arrows: lead pieces (in the neutron CT side-view, the arrow is centred on a region of deep lead corrosion in the upper lead piece); dashed arrows: textile in plug; dotted arrow: loose fragments. X-ray CT data were acquired during the previous study^9^.

**Figure 6:**
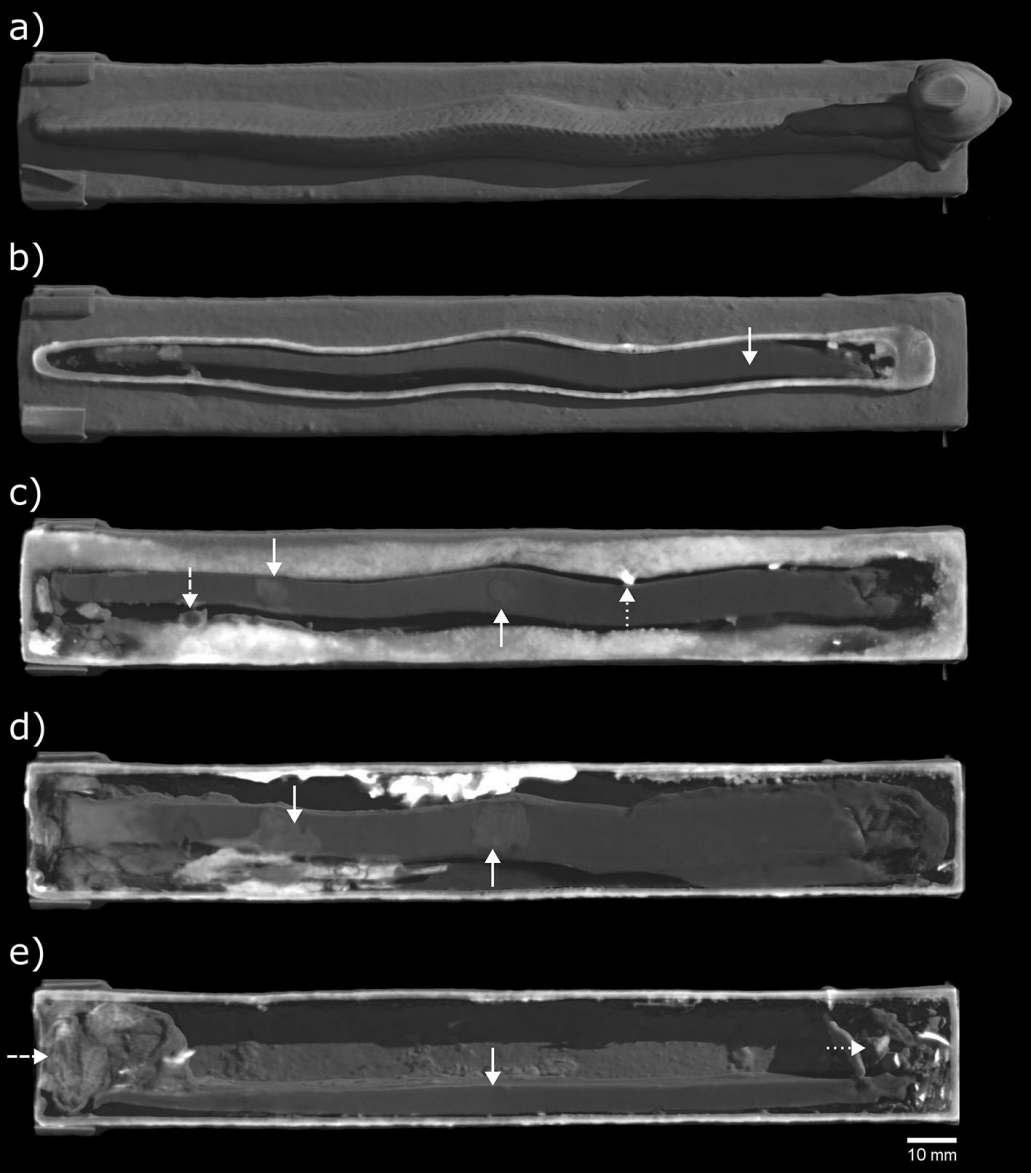
EA71428 neutron CT volume renders. (**a**) complete view of box; (**b**)–(**e**) cutaway views at different heights (measured from the box base): (**b**) 24.0 mm, through eel figure (solid arrow: upper lead piece inside hollow); (**c**) 21.3 mm, through top box wall (solid arrows: lead corrosion, dashed arrow: repair to box, dotted arrow: chaplet); (**d**) 17.7 mm, through base of upper lead piece (solid arrows: lead corrosion); (**e**) 12.9 mm, through box hollow (solid arrow: lower lead piece, dashed arrow: textile in plug, dotted arrow: loose fragments).

Previous X-radiography and X-ray CT scans of box EA71428 uncovered two long objects of high density, each spanning the length of the box interior; neutron tomography shows low attenuation from these objects (0.2–0.4 cm^−1^), suggesting they are made from lead (Fig. 5). The upper lead object (approx. 179 mm long, 13 mm tall) fits inside the eel figure on the box, matching its sinuous form on its upper surface and spreading to a flat, wide base (Figs. 5b and 6). Due to its shape, it is assumed that the upper lead piece was poured into the box in a molten state whilst the box was upside down, and it subsequently detached from the internal box walls once solidified. On the underside of the upper lead piece, across most of its length, is a layer of corrosion which could have originated from proximity to decaying organic material. This corrosion penetrates more deeply into the lead where it is in contact with the plaster and textile plug, and at two points further into the box, where it appears to have spread into the lead from distinct small areas on the surface (Fig. 6c,d). The lower lead object is rectangular in cross section (approx. 166 × 13 × 5 mm), and roughly flat along its length, leaning at an angle against the internal wall of the box (Figs. 5b and 6e). This lower lead object appears to act as a support for the upper, preventing it from falling to the base of the box interior.

A plug containing folded textile is seen immediately inside the square opening at the rear of the box (Figs. 5b and 6e). It is thought that this textile is surrounded by the plaster which can be seen visually on the box exterior behind the damaged metal plate covering the opening. A small amount of fragmentary material (approx. 23 × 23 × 13 mm) is visible at the front end of the box (Fig. 5b). The larger fragments show a strong neutron attenuation of 2.0–3.2 cm^−1^, much higher than that measured for the bones in the other boxes. It is not known what these fragments are, but the attenuation appears to be too strong to be from mineralised bone.

Evidence of the manufacture of box EA71428 seen in neutron tomography supports the conclusions drawn in the previous study^9^: the box and eel part of the figure were hollow cast together; the cobra hood with human crowned head was separately solid cast, and attached to the box with a small support at the back of the cobra hood. These attachments appear to have been made by fusion welding (joining the molten pieces together, sometimes using a molten filler alloy of the same composition as the pieces) or hard soldering (joining using a molten alloy of slightly lower melting temperature and a flux)^37^, since no material of significantly different neutron attenuation is present at the joins. Signs of lost-wax casting of the box are evidenced by corroded metal chaplets which are seen throughout: three in the side walls, one in the base, one in the top (passing through the eel figure). Two detached chaplets are present inside the box, thought to have been originally located near the box opening, in the base and the side wall, respectively. Neutron CT revealed a filled round hole, approx. 3.6 mm in diameter, on the box near a point at which the eel body meets the top of the box (Fig. 6c). The neutron attenuation of the fill material in this hole is similar to that of the lead inside the box; it is possible that this is an ancient repair of a defect which formed during casting.

### EA36151

**Figure 7:**
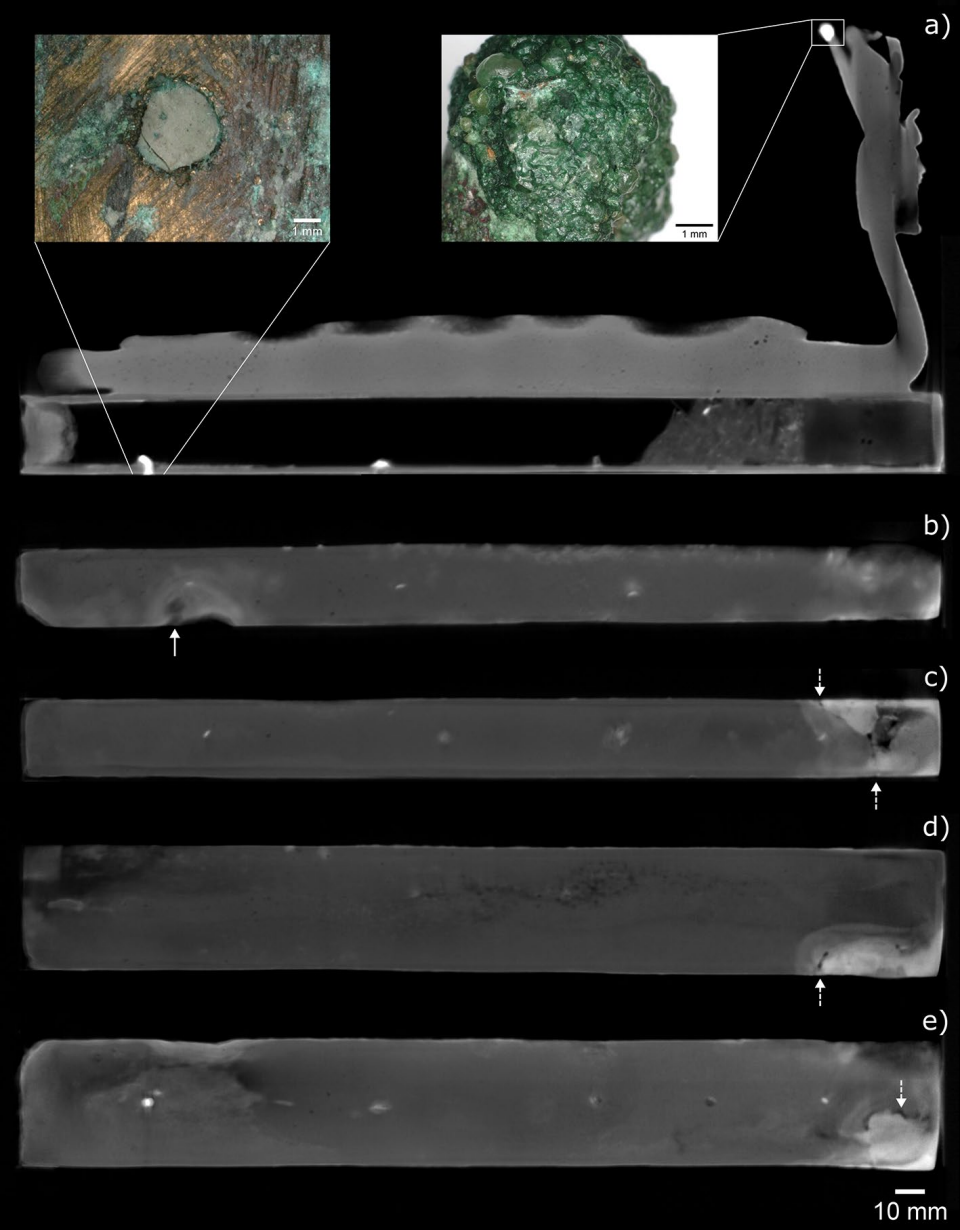
EA36151 neutron CT slices through the (**a**) centre, (**b**) left wall and (**c**) right wall of the box (side-view), (**d**) top and (**e**) base of the box (top-view). Inset microscope images in (**a**) show strongly attenuating material in the base of the box (20× magnification) and the crown of the hybrid figure (50× magnification). Solid arrow in (**b**) indicates an apparent ancient casting repair. Dashed arrows in (**c**)–(**e**) indicate cracks in the box walls, surrounded by strongly-attenuating resin from a past conservation treatment in the British Museum.

Neutron tomography provided evidence for the manufacture, contents and later additions to EA36151, the largest votive box studied for this work. Between the eel figure and the top of the box, a faint boundary is visible in the tomographic data (Fig. 7a). This indicates that the box and figure were cast separately and later joined together, possibly using hard soldering. There are 11 chaplets present in the box remnant from the casting process: four in each side wall, roughly evenly spaced across the length of the box, and three in the base. The attenuation coefficients of the chaplets (1.3–2.0 cm^−1^) are comparable with those in the other five boxes examined in this study, and thus they are also assumed to be corroded iron.

A filled hole of approx. 3 mm diameter is present in the base of the box, where a chaplet may previously have been present. From microscopic imaging of the fill material, it appears to be made from wax, resin or plaster—or a mixture containing multiple components (Fig. 7a). The neutron attenuation of this filler (2.4–2.6 cm^−1^) is markedly greater than for the 11 chaplets. Microscopic imaging and measured neutron attenuation (2.5–2.6 cm^−1^) of the top of the double crown worn by the figure also indicate wax or resin has been applied to this area. It is likely that these two additions to the box were made after its discovery.

Several areas of repair to the box are seen on the neutron CT images. A repair on the left side of the box, appears to have been made during the original manufacturing process, possibly to cover a hole which formed during casting (Fig. 7b). A larger repair, at the front-right corner of the box, is part of a conservation treatment undertaken in the British Museum in 1977: Bondapaste (a polyester resin) was used to repair a region of corrosion and cracking in this corner^38^. Neutron tomography of this corner (Fig. 7c–e) reveals damage in the top, right and base walls, and the relatively high attenuation of the resin (1.4–2.0 cm^−1^) compared with the copper alloy walls (0.6–0.7 cm^−1^). A neutron CT volume render image with microscopy highlighting this conservation treatment is given in Supplementary Information Fig. S3. A region comprising lead is present inside the box, directly behind the repaired surface. The lead contains several rounded voids, up to 5.3 mm wide, and envelops two of the chaplets which penetrate the box interior, implying that the lead was poured into the box in a molten state. It is possible that the lead was added as part of an ancient repair, to provide support to this area of the box.

Box EA36151 also contains loose, fragmented material (approx. 60 × 35 × 22 mm). There are no complete bones that could be identified, although several potential bone fragments are present. The additional presence of highly attenuating objects (1.0–1.6 cm^−1^) in the loose material could indicate soil or sand containing bound water or hydrates. Further neutron CT slice images of the fragments inside box EA36151 are given in Supplementary Information Fig. S4.

A summary of the findings inside the six votive boxes from the neutron CT study is given in Supplementary Information Table S2. Corroded chaplets were found in each box, as summarised in Supplementary Information Fig. S5.

The lead regions found in three of the boxes were segmented  and their volumes calculated. The masses of lead in boxes EA36167, EA71428 and EA36151 were calculated to be 54 ± 3 g, 338 ± 15 g and 351 ± 14 g, respectively, using an assumed density for lead of 11.3 g cm^−3^ (further details in Supplementary Information Table S3); these values are likely to be slight overestimates due to the presence of lead corrosion.

## Discussion

In this work, neutron tomography was utilised to non-invasively study the contents of six votive boxes made of copper alloys from ancient Egypt to search for the presence of faunal remains, and to understand the manufacture of the containers, building on a previous X-ray imaging study. In addition, neutrons were used to identify as lead the dense material present inside three of the six boxes, as previously observed with X-rays. It was difficult to isolate and identify the animal remains in the boxes due to the complexity of the internal assembly and the comparable neutron attenuation of the loose material, textile and plaster also present. Nonetheless, bones were observed in three of the six votive boxes studied (EA27584, EA49146 and EA36167), with possible broken-down bones also present in the larger two boxes (EA71428 and EA36151). Most of the bones are in a fragmentary condition, however complete long bones were observed in boxes EA27584 and EA36167. The neutron CT images also revealed an apparent intact lizard skull inside box EA36167. The dimensions of this skull and the styling of the lizard figure in the casting atop the box are similar to those of lizards of the *Mesalina* genus; however, the variability of skeleton sizes between species/age of lizards makes it difficult to determine the species from the neutron CT scan. Skulls in the remaining boxes were not identified, and it is assumed that either they have broken down over time, or they were not initially present. Textile fragments were observed inside the three boxes in which animal bones were also present, suggesting that the animals were wrapped before being placed inside the boxes.

Within boxes EA36167, EA71428 and EA36151 are significant amounts of lead. Because lead has a much lower melting point than copper and its alloys, the lead must have been placed inside the boxes after they were cast. The shape and distribution of the lead in boxes EA36167 and EA36151 indicate that the lead was molten when introduced to the box, whereas the long, rectangular lower piece in EA71428 was likely solid when inserted. The upper lead piece in EA71428 is probably the result of molten lead being poured into the box whilst it was inverted, since its shape closely follows that of the void inside the eel figure surmounting the box. In ancient Egypt, lead held a magical status and was a material of choice in the manufacture love charms, in rituals of execration of enemies, or, particularly interestingly in our case, in the protection of mummies^39^. Horus eye incision-plates applied over the incision by the embalmer could be made from lead, although other materials are attested. A lead core was also previously discovered inside a bronze falcon figure from Saqqara^40^. It seems that only a small range of Egyptian divine figures or sacred items were regularly made out of lead, perhaps due to symbolic connotations of this material rather than its cheap economic cost (see lead figures of Nefertum and of child deities, as well as models of the Osirian processional barge in lead from Thonis-Heracleion^41^). Neutron tomography of box EA71428 revealed no inscriptions on the surfaces of the lead pieces. It is plausible that the addition of the lead could also have been prompted by practical uses, such as lowering the centre of mass of boxes with tall, solid metal figures at one end, or providing additional support to weak or damaged areas, as may be the case in EA36151. Such explanations would appear less valid for EA36167, however.

Of the six boxes studied in this work, there are loops for suspension on the three boxes without lead inside, and lead is present in each of the three boxes without loops. It is surmised that boxes featuring loops would have been suspended from shrine or temple walls, cult statues or sacred boats used in procession, rather than placed on a surface. Neutron imaging also reveals corrosion on the surface of the lead in boxes EA36167 and EA71428. The higher neutron attenuation seen in the regions of corrosion suggests the presence of hydrogen-containing corrosion products, possibly a result of contact of the lead with the air and decaying animal remains, and the plaster plug in the case of box EA71428.

Evidence for the use of lost-wax casting in the votive box manufacture is seen in the presence of multiple chaplets in each box. The strong neutron-attenuation of the chaplets indicates that they contain hydrogen-bearing corrosion products; it is proposed that iron chaplets were used, since it is a material less resilient to corrosion than copper alloys. The number of chaplets present in each box is roughly proportional to the box dimensions, since they were intended to ensure structural stability of the core materials inside the mould after the wax was melted and removed. Core material remains inside box EA27584, with the chaplets embedded inside it. In most cases, the animals depicted on top of the boxes appear to have been cast together with the box. The largest boxes (EA36151 and EA71428) have features suggesting that part or the entire animal was soldered or fusion welded to the top surface of the box. The variety of techniques used to make the boxes, in addition to the variety in their dimensions, suggests that there was not a standardised production method, although comparable manufacturing techniques seem to have been used for small-sized boxes.

## Conclusion

In this work we show that neutron CT is an effective alternative or complementary technique to X-ray CT for the non-destructive examination of ancient Egyptian copper alloy votive boxes, given their often high lead content, and the presence of lead and/or organic material contained within. While the presence of lead created streaking and beam hardening reconstruction artefacts in X-ray CT^9^, the use of neutrons allowed us to virtually unseal the votive boxes and reveal their organic/low density content, including faunal remains and textile wrappings. Neutron CT also revealed repairs and damage to box EA36151 which were not detected by X-ray CT, due to the proximity of lead to the damaged area, and the low density, but strongly neutron-attenuating later additions in wax or resin. In box EA36167, the lead region obscured the animal remains and wrappings on the X-ray CT scan which were subsequently revealed with neutrons in this work.

This work provides further evidence for the use of copper alloy votive boxes in ancient Egypt, showing that animal remains were wrapped in linen and placed inside the boxes before they were sealed, and that the cast animal figures upon the boxes were potentially intended to correspond to the remains within.

## Methods

Neutron tomography of the votive boxes was conducted on the IMAT beamline at the ISIS pulsed neutron and muon source (Rutherford Appleton Laboratory, UK). IMAT is a cold neutron instrument which captures images of objects based on their neutron attenuation^42–44^. The tomography process on IMAT is similar to that for laboratory-based X-ray CT and synchrotron radiation CT scans: a series of radiographs are acquired throughout a step-by-step rotation about the vertical axis. These projections are used to create a volumetric reconstruction of the object^45^ in which every voxel describes the local neutron attenuation coefficient as a grayscale value.

For our set-up we used a pinhole size of 40 mm with 10 m pinhole-to-sample distance, giving a best achievable resolution of about 100 μm. The votive boxes were mounted with their longest axis aligned vertically. This configuration allowed the boxes to be positioned as close to the detector as possible, minimising image blurring and variations in beam path length through the box during scanning. The mounts were made of aluminium, with Teflon tape used to protect the surface of the boxes. Both these materials have low neutron attenuation coefficients.

Projection images were acquired using an ANDOR Zyla sCMOS 4.2 PLUS camera (2048 × 2048 pixels) coupled with an optical lens and a 100 µm thick ZnS/LiF scintillator sheet. An acquisition time of 30 s per projection was selected for each scan, as a compromise between improved counting statistics and the total time available for the experiment. Each box was rotated through 360° during the scan. The number of projections was selected for each box based on the Nyquist–Shannon theorem—i.e., *S*(π/2), where *S* is the number of horizontal pixels covered by the box at its maximum width throughout the scan. The scan parameters used for each votive box are given in Supplementary Information Table S4.

Flat field (illumination of the detector with the sample outside the field of view) and dark field (with the beam shutter closed) images were acquired for each scan and used to correct the projections for inhomogeneity in the neutron beam intensity, detector pixel response, and camera noise. The projections were corrected using the Fiji distribution of the ImageJ software package^46^. Bright spots on the projections due to high energy gamma interactions were removed using the “remove outliers” function in Fiji.

CT reconstruction was performed in the Octopus Reconstruction software package^47^, using a parallel beam filtered back projection algorithm^48^. Due to the larger size of boxes EA71428 and EA36151, they were scanned in two overlapping vertical sections; the resulting reconstructed volumes were subsequently stitched together using the “Pairwise Stitching” plugin in Fiji^49^. Segmentation and volume rendering of the tomographic datasets was performed using VGStudio MAX 3.3 (Volume Graphics GmbH, Germany). Maximum intensity projection (MIP) images—i.e., two-dimensional visualisations of the highest attenuating voxels across multiple tomographic slices—have been used in this article to highlight three-dimensional features in the boxes, including textiles and metal corrosion. The MIP images were made using the “Z Project” function in Fiji. The volumes of segmented lead pieces were calculated using the “Voxel Counter” plugin in Fiji.

In addition, microscopy of specific details highlighted from the imaging investigation was conducted with a VHX-5000 digital microscope (Keyence, Japan), operated in reflective light mode, without filters.

## Supplementary Information

Supplementary Information.

## Data Availability

The neutron CT raw data generated for this work are available to download from the STFC ISIS Neutron and Muon Source repository: https://doi.org/10.5286/ISIS.E.RB1910562. The reconstructed CT volumes are available to download from the Harvard Dataverse: https://doi.org/10.7910/DVN/RGF7BH. Measured neutron attenuation coefficients for the different regions and materials present in each box are given in Supplementary Information Table S5.
