# Supplementary material for: Neutron tomography of sealed copper alloy animal coffins from ancient Egypt
Source: Sci Rep. 2023 Apr 20;13:4582. doi: 10.1038/s41598-023-30468-4 (PMC10119080; doi:10.1038/s41598-023-30468-4)
Supplement: Supplementary file 1 — Supplementary Information. [file 41598_2023_30468_MOESM1_ESM.docx]

Supplementary Information for
**Neutron tomography of sealed copper alloy animal coffins from ancient Egypt**Daniel O’Flynn ^1,*^, Anna Fedrigo ^2^, Laura Perucchetti ^1^ and Aurélia Masson-Berghoff ^3
1^ Department of Scientific Research, British Museum, Great Russell Street, London WC1B 3DG, UK
^2^ Science and Technology Facilities Council (STFC), ISIS Neutron and Muon Source, Didcot OX11 0QX, UK
^3^ Department of Greece and Rome, British Museum, Great Russell Street, London WC1B 3DG, UK
^*^ **email:** [DOFlynn@britishmuseum.org](mailto:DOFlynn@britishmuseum.org)

**Table S1.** Morphometric measurements from an X-ray micro-CT scan of lizard M. rubropunctata (w: width, l: length, h: height). Scan downloaded from MorphoSource (Ref. 36)

| **Bone/structure** | **Size (mm)** |
| --- | --- |
| Orbit | 3.5 ± 0.1 (w) |
|  | 4.5 ± 0.1 (l) |
| Mandible | 6.7 ± 0.1 (w) |
|  | 10.3 ± 0.1 (l) |
| Humerus | 5.8 ± 0.1 |
| Radius | 4.3 ± 0.1 |
| Ulna | 4.8 ± 0.1 |
| Femur | 7.6 ± 0.1 |
| Tibia | 6.7 ± 0.1 |
| Fibula | 6.4 ± 0.1 |
| C1 vertebra | 2.4 ± 0.1 (h) |
|  | 1.9 ± 0.1 (w) |
|  | 1.6 ± 0.1 (l) |

**Table S2**. Summary of findings from neutron tomography of the votive boxes

| **Accession number** | **Animal remains** | **Textile wrappings** | **Lead** | **Suspension loops** | **Number of chaplets** | **Wall thickness (mm)** |
| --- | --- | --- | --- | --- | --- | --- |
| EA27584 | ✓ | ✓ | 🗶 | ✓ | 3 | 1.3 - 1.9 |
| EA49144 | ? | 🗶 | 🗶 | ✓ | 2 | 1.0 - 2.7 |
| EA49146 | ✓ | ✓ | 🗶 | ✓ | 3 | 1.0 - 2.8 |
| EA36167 | ✓ | ✓ | ✓ | 🗶 | 3 | 0.9 - 2.5 |
| EA71428 | ? | 🗶 | ✓ | 🗶 | 7 | 1.2 - 2.1 |
| EA36151 | ? | 🗶 | ✓ | 🗶 | 11 | 2.4 - 3.1 |

**Table S3.** Calculated lead volumes and masses, assuming a lead density of 11.3 g cm^-3^ (box EA71428 contains two lead objects).

| **Accession number** |  | **Calculated lead volume (cm^3^)** | **Calculated lead mass (g)** | **Measured total box mass (g)** | **Lead fraction of total box mass** |
| --- | --- | --- | --- | --- | --- |
| EA36167 |  | 4.8 ± 0.3 | 54 ± 3 | 151 ± 1 | 0.36 ± 0.03 |
| EA71428 | total | 29.9 ± 1.4 | 338 ± 15 | 604 ± 1 | 0.56 ± 0.03 |
|  | upper | 18.4 ± 0.8 | 208 ± 10 |  |  |
|  | lower | 11.5 ± 0.5 | 130 ± 6 |  |  |
| EA36151 |  | 31.1 ± 1.2 | 351 ± 14 | 1597 ± 1 | 0.22 ± 0.01 |

**Table S4.** Neutron CT scan parameters. In each scan, the counting time was 30 seconds per projection. (*EA36151 and EA71428 were scanned in two vertical sections, which were stitched together after CT reconstruction).

| **Accession number** | **Number of projections** | **Voxel size (mm)** | **Lens (mm)** | **Field of View**  **(mm)** |
| --- | --- | --- | --- | --- |
| EA27584 | 1076 | 0.055 | 85 | 113×113 |
| EA49144 | 675 | 0.055 | 85 | 113×113 |
| EA49146 | 758 | 0.055 | 85 | 113×113 |
| EA36167 | 873 | 0.055 | 85 | 113×113 |
| EA71428 | 1443 × 2* | 0.055 | 85 | 113×113 |
| EA36151 | 850 × 2* | 0.103 | 50 | 211×211 |

**Table S5.** Neutron linear attenuation coefficients (in cm-1) measured at different regions of the votive boxes under investigation

| **Accession number** | **Box (subsurface metal)** | | **Box (surface)** | **Lead** | **Lead corrosion** | **Core material** | **Plaster plug** | **Chaplets** | **Bone** | **Fragments (bone?)** | **Textile** | **Strongly attenuating loose fragments** | **Repairs (resin and unknown material)** |
| --- | --- | --- | --- | --- | --- | --- | --- | --- | --- | --- | --- | --- | --- |
| EA27584 | | 0.7–0.8 | 1.1–1.3 | - | - | 0.2–0.3 | 0.5–0.6 | 1.5–1.6 | 0.4–0.5 | 0.2–0.4 | 0.7–0.8 | - | - |
| EA49144 | | 0.7–0.8 | 1.5–1.8 | - | - | - | 0.3–0.5 | 1.4–1.6 | - | - | 0.6–1.0 | - | - |
| EA49146 | | 0.7–0.8 | 1.2–1.8 | - | - | - | 0.4–0.5 | 1.8–2.1 | 0.3–0.5 | 0.3–0.5 | - | - | - |
| EA36167 | | 0.7–1.0 | 1.2–1.8 | 0.2–0.3 | 1.5–1.8 | - | 0.7–1.0 | 1.5–1.6 | 0.4–0.8 | 0.4–0.6 | 0.5–0.8 | 1.0–1.2 | - |
| EA71428 | | 0.6–0.8 | 1.0–1.3 | 0.2–0.4 | 0.4–0.7 | - | 0.6–0.9 | 1.6–2.1 | - | 0.3–0.6 | 0.5–0.9 | 2.0–3.2 | - |
| EA36151 | | 0.6–0.7 | 0.8–1.0 | 0.1–0.3 | - | - | 0.6–0.8 | 1.3–2.0 | - | 0.3–0.5 | - | 1.4–1.6 | 1.4–2.6 |


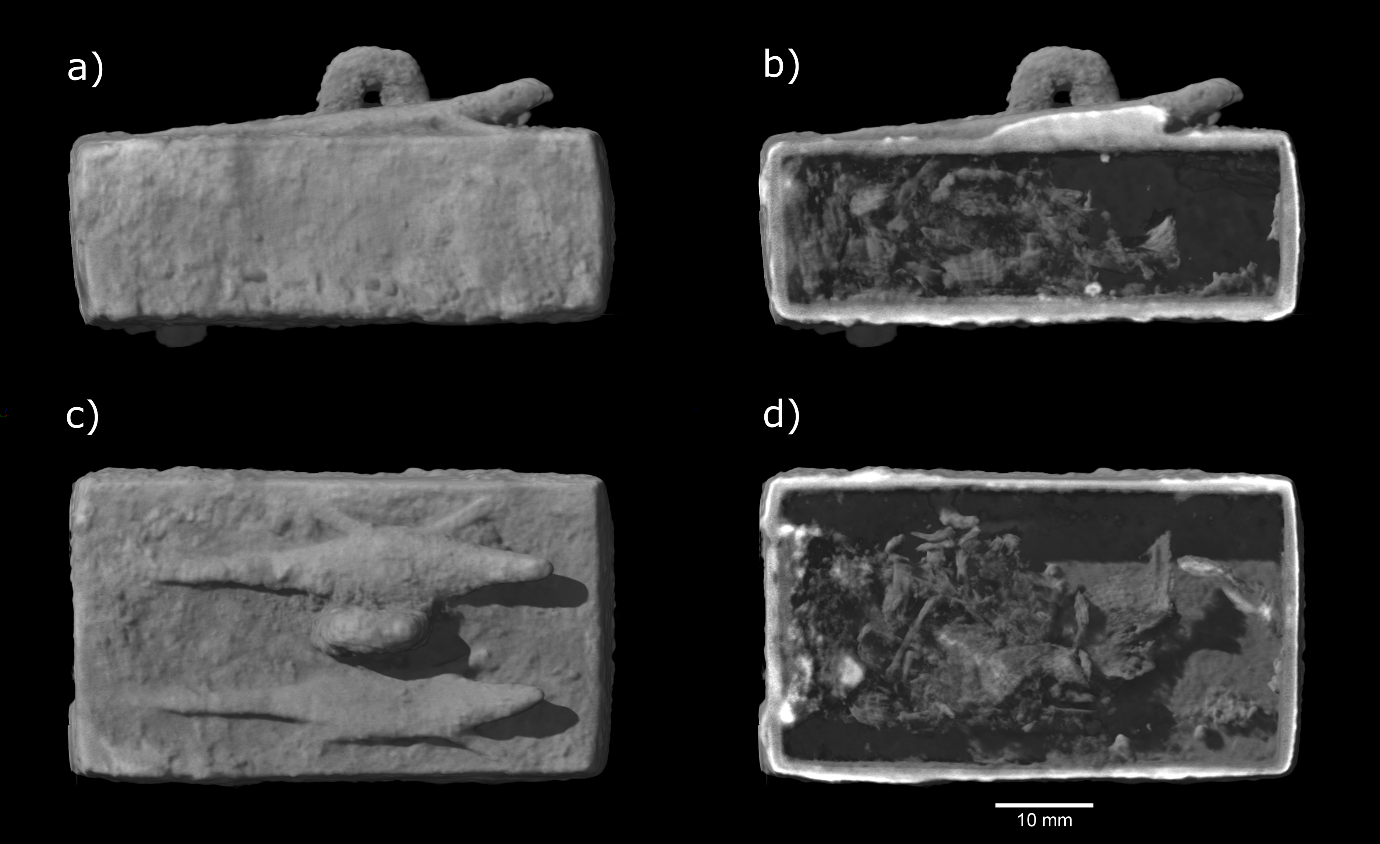


**Figure S1.** EA27584 neutron CT volume renders: **a)** side-view; **b)** virtual cutaway using a vertical plane showing textile remains inside box; **c)** top-view; **d)** virtual cutaway using a horizontal plane.


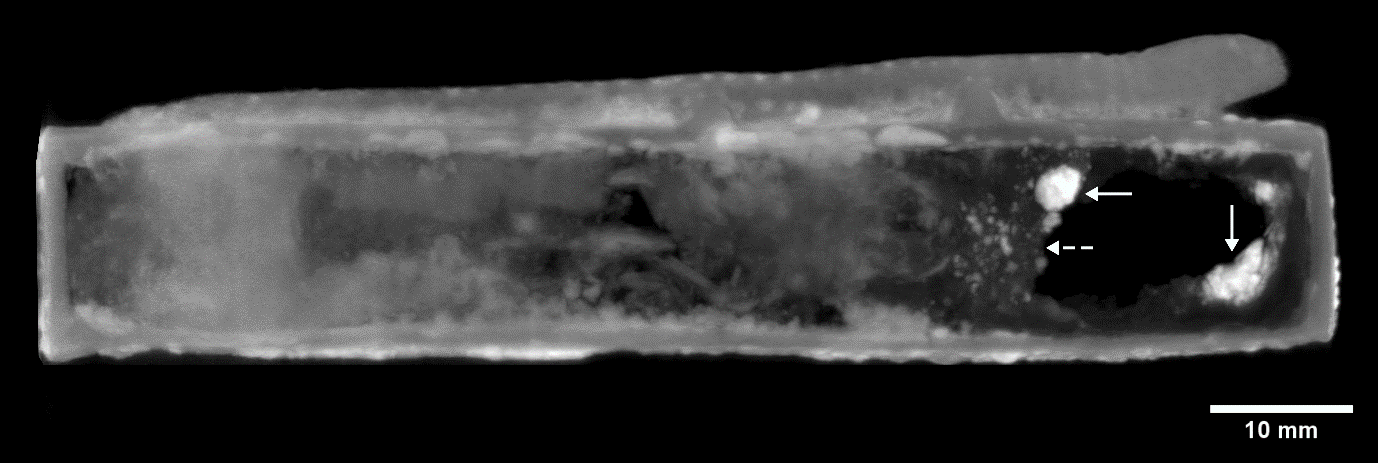


**Figure S2.** EA36167 multiplanar MIP neutron CT image (side-view, 5.56 mm slice thickness, slice centred 15.3 mm from right side of box). Solid arrows: lead corrosion; dashed arrow: a region of concretion between the lead and the wrapped animal remains.


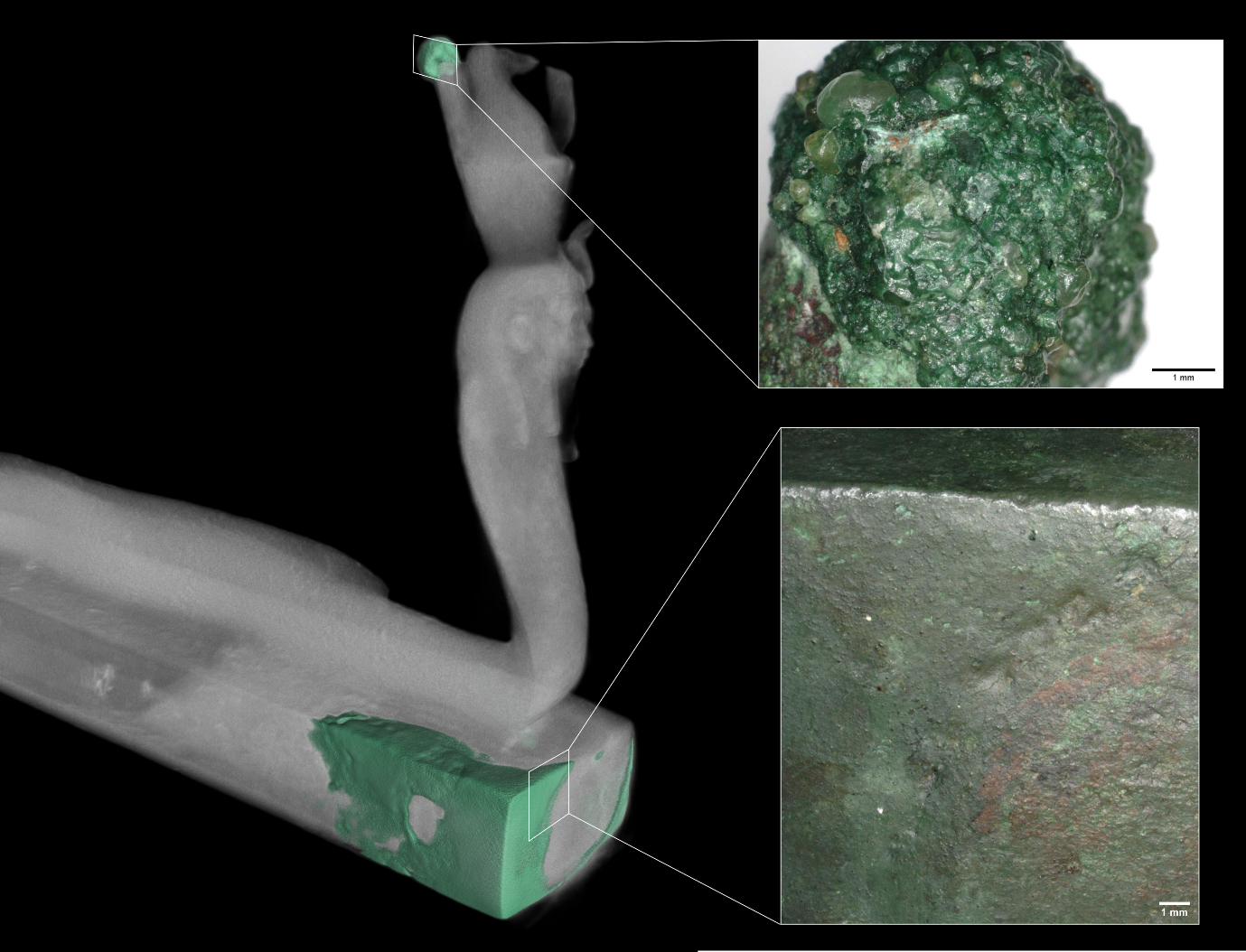


**Figure S3.** EA36151 neutron CT volume render, with segmented green regions indicating regions of high neutron attenuation, and inset microscope images. Upper inset: crown of figure at 50× magnification, showing the presence of apparent wax or resin. Lower inset: front of box at 20× magnification, showing resin from a past conservation treatment in the British Museum.


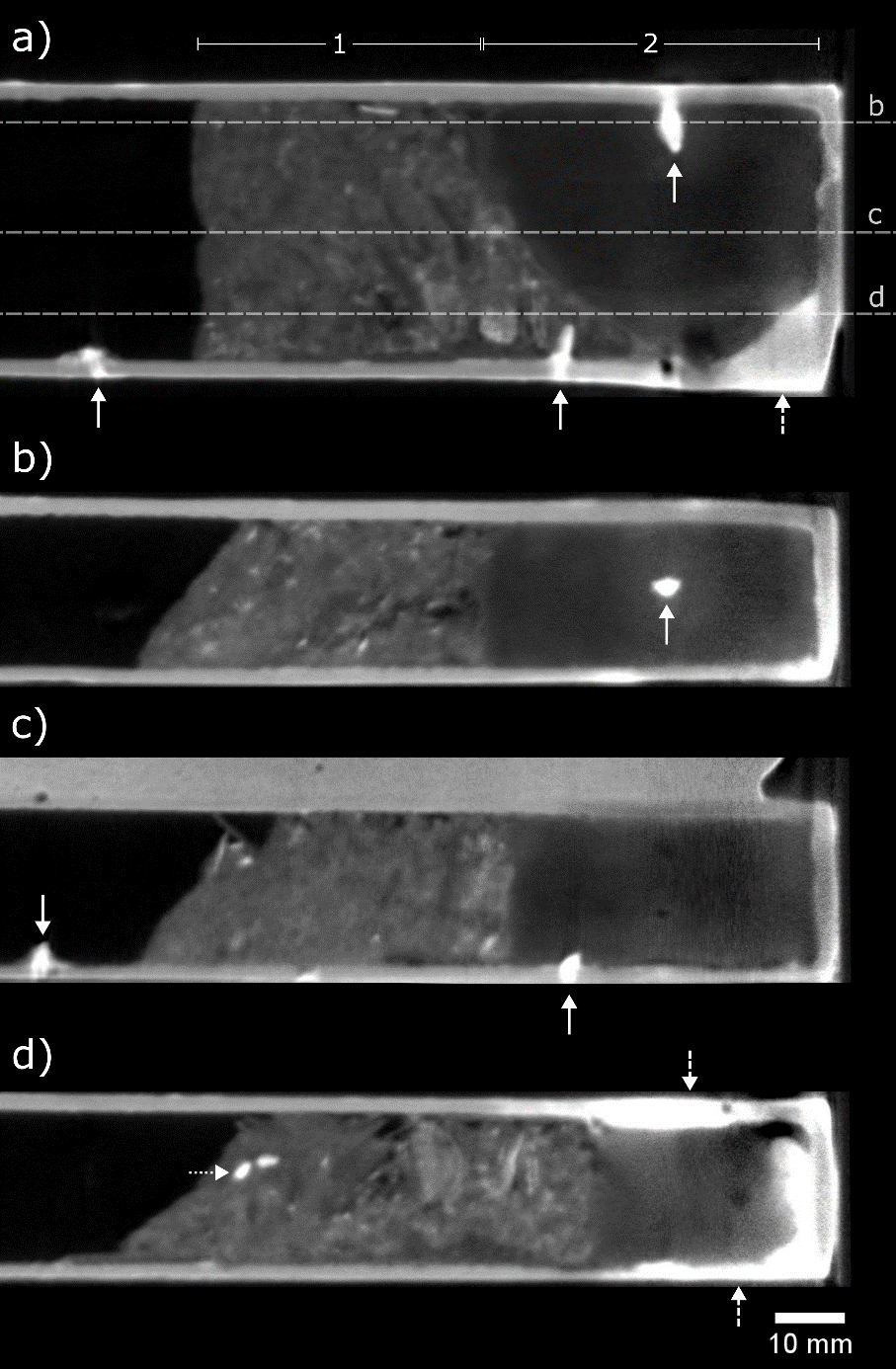


**Figure S4.** EA36151 neutron CT slices. **a)** top-view, 14.0 mm from base of box, showing two internal regions: **1** loose fragments; **2** lead. **b)-d)** side-views, at positions indicated by the grey dashed lines in a). Solid arrows: chaplets; dashed arrows: strongly-attenuating resin-based repairs; dotted arrow: possible bound water or hydrates.


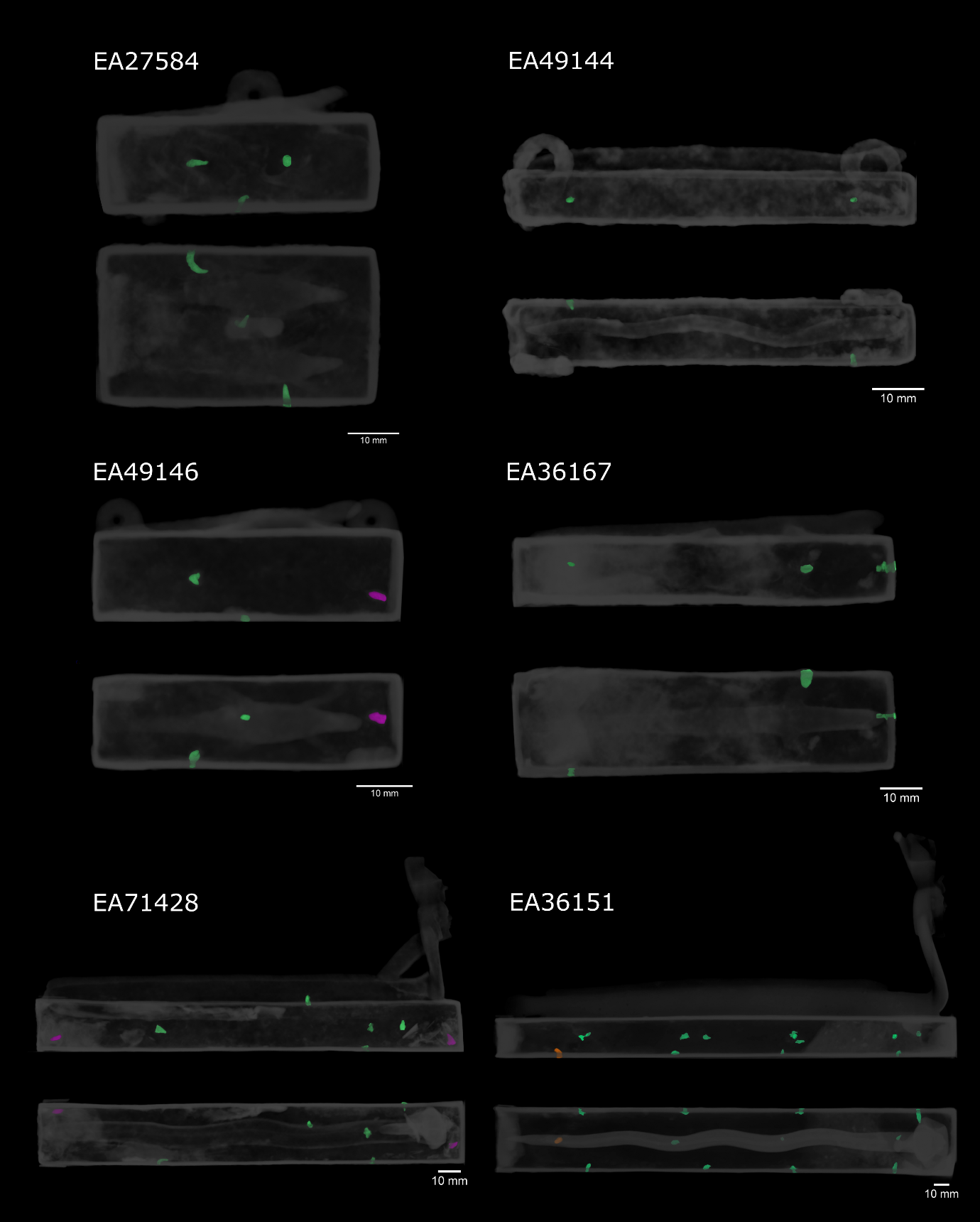


**Figure S5.** The chaplet distributions in the votive boxes (side and top-views, not to scale). The green-coloured chaplets are still embedded in the walls of the boxes; the magenta-coloured chaplets have subsequently broken off; the orange-coloured material in box EA36151 is a later addition, possibly made from wax, resin, and/or plaster.
